# The phenomenology of deep brain stimulation-induced changes in OCD: an enactive affordance-based model

**DOI:** 10.3389/fnhum.2013.00653

**Published:** 2013-10-10

**Authors:** Sanneke de Haan, Erik Rietveld, Martin Stokhof, Damiaan Denys

**Affiliations:** ^1^Department of Psychiatry, Academic Medical Center, University of AmsterdamAmsterdam, Netherlands; ^2^Cognitive Science Center Amsterdam, University of AmsterdamAmsterdam, Netherlands; ^3^Department of Philosophy, Institute for Logic, Language and Computation, University of AmsterdamAmsterdam, Netherlands; ^4^The Netherlands Institute for Neuroscience, Royal Netherlands Academy of Arts and SciencesAmsterdam, Netherlands

**Keywords:** Phenomenology, psychiatry, deep brain stimulation, obsessive-compulsive disorder, affordances, enactivism, qualitative research, neurophenomenology

## Abstract

People suffering from Obsessive-Compulsive Disorder (OCD) do things they do not want to do, and/or they think things they do not want to think. In about 10% of OCD patients, none of the available treatment options is effective. A small group of these patients is currently being treated with deep brain stimulation (DBS). DBS involves the implantation of electrodes in the brain. These electrodes give a continuous electrical pulse to the brain area in which they are implanted. It turns out that patients may experience profound changes as a result of DBS treatment. It is not just the symptoms that change; patients rather seem to experience a different way of being in the world. These global effects are insufficiently captured by traditional psychiatric scales, which mainly consist of behavioral measures of the severity of the symptoms. In this article we aim to capture the changes in the patients' phenomenology and make sense of the broad range of changes they report. For that we introduce an enactive, affordance-based model that fleshes out the dynamic interactions between person and world in four aspects. The first aspect is the patients' experience of the world. We propose to specify the patients' world in terms of a field of affordances, with the three dimensions of broadness of scope (“width” of the field), temporal horizon (“depth”), and relevance of the perceived affordances (“height”). The second aspect is the person-side of the interaction, that is, the patients' self-experience, notably their moods and feelings. Thirdly, we point to the different characteristics of the way in which patients relate to the world. And lastly, the existential stance refers to the stance that patients take toward the changes they experience: the second-order evaluative relation to their interactions and themselves. With our model we intend to specify the notion of being in the world in order to do justice to the phenomenological effects of DBS treatment.

## Introduction

People suffering from Obsessive-Compulsive Disorder (OCD) do things they do not want to do, such as endlessly washing their hands, checking the oven, counting their steps, or tidying up their room. And/or they think things they do not want to think: they are plagued by aggressive, sexual, or blasphemous visions. Some people picture themselves hurting their own children, others think they spread deadly germs. In about 10% of these patients, none of the available treatment options [pharmacotherapy or cognitive behavioral therapy (CBT)] is effective. The disease becomes chronic and often the patient's situation deteriorates. Patients may hardly leave their house anymore, or even their room, or even their bed. A small group of these patients is currently being treated with deep brain stimulation (DBS). DBS involves the implantation of electrodes in the brain. These electrodes give a continuous electrical pulse to the brain area in which they are implanted (in the case of this study, the nucleus accumbens), as a kind of pacemaker in the brain.

It turns out that patients may experience profound changes, either immediately, i.e., the seconds after the DBS is turned on, or at a later stage, when the optimal settings of the electrodes have been found. Immediate effects for instance are that patients become emotional, or they report seeing colors more brightly, or feeling “as if the shutters have been opened,” or experiencing themselves as equals of the doctors that they had been looking up to just a minute ago. Long term effects include changes in self-esteem, in social interest and communicative interactions, an increase of spontaneous actions, increased expressiveness and creativity, and even the experience of being oneself again.

These global and profound changes are insufficiently captured by traditional psychiatric scales, which mainly consist of behavioral measures of the severity of the symptoms. In fact, some patients show only little improvement on the standard OCD scale, whereas they experience a difference of day and night: to them, life has become liveable again. It is thus not just the symptoms that change. These patients rather seem to experience a different “way of being in the world” (Heidegger, 1927/1978).

Traditional scales fall short to capture these changes. But the patients' experiences are highly relevant: after all, they form both the starting point for understanding and categorizing their problems, and for determining treatment options, as well as the ultimate criteria for evaluating the success of treatment. Psychiatry cannot get round accounting for patients' experiences^1^. In this case, to understand the impact of DBS treatment we need to find a way to make sense of the wide range of changes that patients report.

The methods and concepts as developed in phenomenology may be of particular use to account for the patients' experiences. The phenomenological method requires us to start from the patients' actual experience, rather than starting from theory or from established categories (such as for instance presented by the DSM IV). Moreover, it aims to get beyond merely describing individual changes in order to elucidate changes in the *structure* of experiences. In our research project we combine qualitative interviews with phenomenological analysis. The interviews serve to get an overview of the patients' experiences. Phenomenological analysis in turn helps to distinguish common patterns, or structural changes among the wide range of individual changes. The phenomenological tradition also provides a set of concepts that are helpful to articulate (pre-reflective) experiences.

This paper is primarily a philosophical paper. Based on our findings so far, we have developed an enactive affordance-based^2^ model that is intended to make the changed way of being in the world of the patients more tangible. The model is based on insights from phenomenology, enactivism, and ecological psychology. We will introduce four aspects of the interactions between person and world that together help to make sense of the broad range of changes. Profound changes of course also occur through other forms of treatment, as well as with patients suffering from different psychiatric disorders. The advantage of developing this model with regard to DBS treatment is that, compared to psychotherapeutic and chemical forms of treatment, the effects of DBS can be very quick and direct. This provides a unique opportunity to witness “*in vivo*” the phenomenological changes from disorder to recovery.

In this paper, we will first introduce OCD: its classification criteria as well as some of the relevant phenomenological characteristics. We will then provide a short explanation of DBS, before turning to the phenomenological effects of the DBS treatment. In the subsequent section we present our model and flesh out the four aspects that we deem central to shed light upon the patients' changed way of being in the world. We end with a short discussion of what the effectiveness of DBS implies for our understanding of the relation between neural activity and experiences.

## What is obsessive-compulsive disorder?

John: ‘My whole day is spent checking that nothing will go wrong. It takes me an hour to get out of the house in the morning, because I am never sure that I've turned off all the electrical appliances like the cooker, and locked all the windows. Then I check to see that the gas fire is off five times, but if it doesn't feel right I have to do the whole thing again. In the end, I ask my partner to check it all for me again anyway. At work I am always behind as I go through everything several times in case I have made a mistake. If I don't check I feel so worried I can't bear it. It's ridiculous I know, but I think if something awful did happen, I'd be to blame.’ (Royal College of Psychatrists, 2009).

### Obsessions and compulsions

The fourth edition of the Diagnostic and Statistical Manual of Mental Disorders (DSM IV) defines *obsessions* as recurrent and persistent thoughts, impulses, or images that are experienced as intrusive and inappropriate and that cause marked anxiety or distress (American Psychiatric Association, 2000). Moreover, the person attempts to ignore or suppress such thoughts, impulses, or images, or to neutralize them with some other thought or action. Also, the person recognizes that the obsessional thoughts, impulses, or images are a product of his or her own mind (thus not imposed from without as in schizophrenic thought insertion) (American Psychiatric Association, 2000).

According to the traditional model of OCD, people are first beset by such unwanted thoughts, which causes anxiety, and which in turn leads to *compulsions* as attempts to tame this anxiety^3^. In order to keep control and to avert the lurking danger, people start to develop rituals, such as washing their hands, to wash off the unwanted thoughts, or repeating little prayers as “anti-dotes” to the venomous images. These rituals degenerate into compulsions. DSM IV defines compulsions as “repetitive behaviors (e.g., hand washing, ordering, checking) or mental acts (e.g., praying, counting, repeating words silently) that the person feels driven to perform.” These acts and behaviors are aimed at preventing or reducing distress or at preventing some dreaded event or situation, but they are either “not connected in a realistic way with what they are designed to prevent, or they are clearly excessive” (American Psychiatric Association, 2000).

### Phenomenology of OCD

From a phenomenological perspective, several characteristics are central. First of all, patients suffering from OCD do things they do not *want* to do, and they think things they do not *want* to think. In a basic way they do experience agency: there is no doubt for them that their thoughts are their own, or that they themselves are the ones washing their hands, for instance. This distinguishes them from schizophrenic patients whose basic sense of agency may be disturbed: schizophrenic patients may not experience themselves as the source of their thinking or acting. In another way, however, the agency of OCD patients is impaired: their compulsions are not something they want to do. OCD patients just feel overridingly *driven* to act that way. In this sense, their actions go against ordinary, self-governed agency. This is where the patients' feeling of impaired freedom comes from. Many OCD patients compare their experiences to being imprisoned by their thoughts and/or the constant tension and pressure to perform certain actions.

Secondly, they *know* their compulsive behaviors do not make sense. They know that their behavior is irrational—albeit in different degrees of conviction. Some patients say things like: “I know a bit of dirt won't kill me, but better be safe than sorry,” or: “I know it is ridiculous to wash my hands that often. Still washing your hands is important; you never know what germs you may contaminate your house with.” Others clearly distance themselves from their compulsions, regarding them as downright pointless and nonsensical. In contrast to patients suffering from delusions, OCD patients have *insight* in the fact of the matter, but these facts do not change their feelings of fear and disgust. Their affective states thus seem to take precedence over their rational thoughts.

When patients explain why they do things that they do not want to do and that they moreover consider to be nonsense, they refer to the experience of *tension* and anxiety that becomes unbearable unless they do succumb to performing their compulsive rituals. This tension is so strong that some patients compare this experience to being pushed under water: you HAVE to get to the surface to get air NOW. They describe an extremely anxious feeling when they cannot perform their compulsions immediately—and often they will later make up for the missed rituals. Moreover, patients report that they have to keep on performing their compulsion “*until it feels right.*” This feeling is often described in terms of a lessening of tension, or as a feeling of “*completion*.” That is, only after several repetitions (or hours of repetitions) does the feeling set in that the action is really completed (Pitman, 1987; Rasmussen and Eisen, 1992; Summerfeldt, 2004). One of our patients (P6) described it as the feeling that “the penny has dropped.”

Another remarkable characteristic is the patients' extensive need and *striving for explicit control*. In part, we can understand this need for control as the corollary of the experience of tension. The experience of a certain tension, and of uncertainty whether or not you have done something and whether or not you have done it correctly, is familiar to all of us. Just as familiar is the response to maximize conscious control over your actions.

Speaking very generally, we can say that we experience a certain tension when things are not optimal. According to Merleau-Ponty (1945/2002), our normal actions have the structure of striving toward an optimal grip. Deviations from such optimal grip lead to tension, and to what Rietveld calls “the urge to move to improve” (Rietveld, 2012a). Merleau-Ponty gives the example of moving to the optimal distance to watch a painting, Dreyfus (Dreyfus, 2002) mentions the optimal distance toward other people in an elevator. Attaining and maintaining an optimal grip usually happens quite unreflectively. This specific distance just “feels right”: it is not so much that we pay conscious attention to, or deliberate on our acts, but rather, in a way that resembles skillful improvisation. The tendency toward an optimal grip is a basic concern of living organisms. It shapes the person's selective openness to affordances so that certain affordances stand out as relevant and the individual can unreflectively improve his or her situation by simply being responsive to this structured field of relevant affordances (Rietveld, 2012a,b).

On the other hand, we sometimes do need to deliberate or explicitly pay attention to what we do when our unreflective handling does not suffice. For instance, when you are unsure whether you did something correctly, such as locking the door, you check it. Or when you want to avoid making a mistake, you pay extra, conscious attention to what you are doing. In general, when things go wrong, or when you are learning a new skill, you will rely on deliberation and conscious attention to your acts. The conscious attention to actions is thus not in itself pathological, or even irrational. It is a natural reaction to the experience of tension. It only becomes pathological when the awareness is out of proportion^4^. It seems that obtaining an optimal grip normally implies a balance between unreflective action on the one hand and deliberation and reflexive awareness of one's actions on the other hand.

This balance seems to be disturbed in OCD patients. We suggest that one of the processes that maintains and aggravates this imbalance is what we call the “*hyper-reflexivity trap*” (De Haan et al., 2013). *Hyper-reflexivity* (Frankl, 1946/2009; Laing, 1960/1990; Sass and Parnas, 2003; Fuchs, 2011b) refers to the exaggerated tendency to reflect on or pay conscious awareness to what one is doing—not afterwards (like ruminations) but rather during the performance itself. Every act becomes a conscious, deliberate decision instead of just a spontaneous response to a situation. For example, when you are ill at ease at a reception where you hardly know anybody, you may become unpleasantly aware of yourself, in this room full of people, and you may start to wonder what to do, whether to get another drink, which group to join, how to hold your hands, how to pose yourself. Such awkward self-awareness is the opposite of the fluent, spontaneous way in which we usually deal with the world. In our terminology: the disturbed unreflective way of attaining optimal grip comes with an excessive amount of reflective deliberation.

As Fuchs (Fuchs, 2011b) points out, an experience of alienation of the familiar leads to hyper-reflexivity, but hyper-reflexivity in turn aggravates the alienation. In the case of OCD patients, we can indeed see such a negative spiral at work. The “hyper-reflexivity trap” proceeds through several stages (De Haan et al., 2013):

1. First, there is the feeling of tension: the feeling of having insufficient grip.
2. This feeling leads to attempts to regain control through deliberation (What might have gone wrong? What might go wrong in the future? How can I prevent that?), and reflexive awareness of one's actions (trying to perform all actions with maximal attention).
3. But too much reflexive awareness can be dangerous: analyzing and paying attention to your movements may lead to alienation and typically *augments* insecurity^5^.
4. As a last step, the increase of insecurity brings us back to the first step.

Too much deliberation and reflexive awareness thus disturbs the balance that is required for experiencing grip, and it may even lead to a negative self-reinforcing spiral of feelings of tension and insecurity and subsequent attempts to gain explicit control. Normally we unreflectively know how much deliberation is optimal in particular circumstances and are unreflectively responsive to the experienced tensions to improve our grip on the situation. In OCD a disturbance of the normal tension-guided tendency toward an optimal grip (stage 1) probably sets the hyper-reflexivity trap in motion.

### Suffering and treatment

OCD is a highly disabling disorder. The World Health Organization lists it as one of the twenty most disabling diseases (Heyman et al., 2006). Patients spend much time and energy on their compulsions, which leads to an increasingly small world. They simply lack the time to participate normally in daily life. Moreover, the patients' awareness of the meaninglessness of their behavior adds considerably to their suffering. “If I lift and replace that chair 3000 times each day, then that is a day, well, one might just as well have been dead,” one of our patient (P4) says. Another patient (P1) says: “My life feels so terribly empty (…). Often, when I go to bed at night, I think, “what did I do today?” Well, I have washed my hands, I have cleaned my eyes, I have put on clothes, I have brushed my teeth, I have washed my hair, I have eaten something—and well, that is it.” OCD patients know that their obsessions and compulsions do not make sense and they are often ashamed of what they cannot help but doing. Because of these feelings of shame, they usually try to hide their compulsions from others, and make up all kinds of excuses to avoid situations that trigger these compulsions. But the excuses, the concealing and the lying in their turn only contribute to the aversion they feel toward themselves. In addition, their experience of a lack of understanding by people in their environment often leads to feelings of intense loneliness.

Many patients only start to seek help after many years of increasing difficulties to participate in ordinary life (Heyman et al., 2006). This is perhaps due to the fact that the illness is still relatively unknown, and maybe also because of the shame that patients experience. Once they do seek help, about 50–60% of patients respond well to treatment with Cognitive Behavioral Therapy (CBT) and/or medication (Heyman et al., 2006; Denys et al., 2010). About 10% of these patients, however, do not respond to any form of treatment (Denys et al., 2010). OCD then becomes a chronic disorder. For these patients, DBS may be a treatment option.

## What is deep brain stimulation?

DBS involves the implantation of electrodes in the brain which give a continuous electrical pulse to modulate specific brain areas. Usually the target brain area is stimulated in both hemispheres. Each lead contains four electrodes. The leads are connected through a wire to a battery that is placed subcutaneously below the clavicle or in the abdomen. The activity of the electrode can be programmed externally with a portable appliance communicating with the pulse generator through telemetry. The electrodes can be stimulated separately, as a result of which the anatomic reach of the stimulation area can be adjusted. Frequency, intensity, and pulse width are also programmable. Typical stimulation parameters vary for the frequencies between 2 and 185 Hz, for the current power between 0 and 10 V, and for the pulse widths between 60 and 150 ms. The programming facility has the advantage that, after implantation, the stimulation can be optimized in order to increase the therapeutic effect and to decrease side effects.

DBS is mainly used for people who suffer from advanced Parkinson's disease, but it is also used as a treatment against epilepsy, movement disorders, dystonia, and chronic pain. About 80.000 people worldwide have been treated with DBS, the majority of which are Parkinson's disease patients. In the last decade, the use of DBS for treatment resistant psychiatric disorders is being tested (Mayberg et al., 2005; Mallet et al., 2008; Malone et al., 2009; Denys et al., 2010). There are no exact figures on the number of psychiatric patients who are treated with DBS. We estimate that worldwide ~200 patients with psychiatric disorders receive DBS treatment. At the Academic Medical Center (AMC) in Amsterdam 42 OCD patients and 22 patients with major depression are currently being treated. That makes it one of the biggest centers for DBS treatment for psychiatric disorders.

Since DBS is still a new and experimental form of treatment for psychiatric disorders, there is no consensus yet on which brain region would be the best target for the various disorders (de Koning et al., 2011). At the AMC, both OCD patients and major depression patients are stimulated at the nucleus accumbens. An earlier study has shown that this treatment is effective in ~2/3 of the OCD patients (Denys et al., 2010).

Until now, the exact operating mechanism of DBS is not known. There are two general hypotheses. (McIntyre et al., 2004). One is that DBS causes a functional lesion by *inhibiting* the stimulated brain core. This inhibition can be caused by a depolarization blockage of the neurons, by synaptic depression (exhaustion) or by synaptic inhibition via “neuronal jamming”; inducing a meaningless activation pattern (McIntyre et al., 2004). The second hypothesis is that DBS *activates* the neuronal network connected to the brain core which is stimulated. Then stimulation leads to a modulation of the pathological activity in the neuronal network. It is most likely that the therapeutic effects of DBS are caused by a combination of direct and indirect effects dependent on the specific cytoarchitecture of the stimulated brain area. Because the field intensity of the electrode decreases exponentially with distance, neurons are influenced in various ways. The neuronal cell body is probably inhibited in the center of the stimulation area, and the axonal terminals are stimulated on the edge of the stimulation area.

Based on fMRI and EEG research with OCD patients who are stimulated in the nucleus accumbens, the AMC group hypothesizes that DBS causes changes in the *connectivity* between brain areas and restores intrinsic brain network dynamics, rather than merely having inhibitory or excitatory effects at the target area (Figee et al., 2013). In particular, stimulation of the nucleus accumbens of OCD patients “normalizes nucleus accumbens activity and restores intrinsic frontostriatal network dynamics” (Figee et al., 2013). Moreover, they found that frontostriatal connectivity changes were strongly correlated with OCD symptom improvement. It seems that DBS normalizes pathological brain functioning locally at the stimulation site and globally throughout its connected frontostriatal network (Figee et al., 2013).

## Effects of DBS treatment on the experiences of OCD patients

### Methods

We are currently investigating the effects of DBS treatment on the OCD patients' experiences by combining with qualitative research methods with phenomenologically informed analysis. Qualitative research methods are aimed at answering questions of “how” and “why,” in contrast to the “how much” questions that drive quantitative research (Boeije, 2010). Qualitative research methods are especially well-suited to investigate little known phenomena, that is, to map a phenomenon in all its complexities. Additionally, on the basis of such rich mappings, questions with regard to frequency can be developed. In general, qualitative research generates hypotheses, which can be tested further, also with quantitative research means. Besides, qualitative research methods are particularly suited to investigate personal experiences without distorting them by forcing them into pre-defined categories. Given that the effects of DBS treatment for psychiatric patients are still a relatively unknown territory, and that our aim is to get a grip specifically on the patients' experiences, qualitative research lends itself well for our project.

Our research roughly follows the “grounded theory approach” (Strauss and Corbin, 1994). That is, the data collection is primary and we develop our theory along the way of making sense of these data. Instead of starting research with a theory and testing it through the collection of data, the theory is developed by interpreting the data, from “bottom-up.” As is the usual practice in qualitative research, data collection and data analysis are continuously alternated. Our research is a loose variant of this approach, because we do bring in our own specific expertise in phenomenology and ecological psychology, which inevitably shapes our analysis and the kinds of concepts that we use. Our analysis is thus phenomenologically inspired in the sense that we mainly draw on phenomenological concepts in developing a theory out of the data. In this philosophical paper, we focus on presenting the model that we have developed out of our data so far.

Of the wide range of qualitative research methods, we have used semi-structured interviews. These interviews consist of broad, open questions in order to elicit, and stay as close as possible to, the patients' own experiences. They are semi-structured, which means that the interviewer has an (evolving) list of topics to be covered. If those topics are not brought up by the patient, the interviewer will inquire after them—again using only open questions. The interviews start by asking patients to sketch their personal background, and the course of their disorder. After this introduction, the main question is asked: “what has changed as a result of DBS treatment?” The topics are divided into three sections. The section “person” consists of questions on changes with regard to body and movement, perception, emotion, cognition, action, interests, and abilities. The section “social” explores changes in social interactions and relationships. And lastly, there is a section on existential questions pertaining the patients' relation to themselves, and to their surroundings, their stance on their disorder, and their experiences of freedom and time.

### Patients

This study is based on interviews (by the first author) with 14 patients, all of whom are treated with DBS in the nucleus accumbens, and receive, or have received, CBT. The sample consisted of 7 women and 7 men, ranging in age from 25 to 67 at the time of the interview. The patients are at various stages of treatment: some have been stable for years, while others are still in the initial phase of finding the right DBS settings.

### Preliminary results

It turns out that when the DBS is effective the phenomenological effects are both global and thorough. We now give a selection of quotes to illustrate the wide range of phenomenological changes that occur.

Some patients **immediately** experience effects after switching on the battery:

‘I am back again: I can feel myself again. I can feel the ground. (…) The colours are brighter. (…) Before there was just fear, now I am back again.’ (P7)

‘It is as if you are very much in love all of a sudden. (…) As if you become much lighter somehow, but in very pleasant way.’ (P1)

‘I felt very emotional and I felt I was becoming happier. Yes, and less anxious. (…) And somewhat more indifferent. Like: What do I care?’ (P2)

‘When the DBS was turned on, that was very strange! I had this pleasant feeling all of a sudden. (…) Right before, I had been quite negative and then, two minutes later, I was thriving. (…) Before I saw all the restrictions, you know, from having this disorder. All the things I cannot do. (…) But after the DBS was turned on, I thought: “oh but I *can* do this”.’ (P3)

‘I just started talking, also to all those doctors around the table, and I didn't look up to them anymore, I just regarded them as ordinary humans, you know.’ (P14)

Patients often describe their changed experience in terms of **mood**:

‘With the right settings, I am in a better mood. I can immediately notice that. It's … my breathing is calmer and more relaxed. (…) I feel less tense and so, well, I have less the need to do these compulsive actions’ (P3)

‘The change in my mood, in my feeling of hope and courage, is very noticeable in my urge to make plans. (…) To think beyond where you are at the moment.’ (P1)

**Long-term effects** include spontaneous actions, changes in social interactions, the ability to enjoy, and even the experience of being oneself:

‘It is as if the shutters have been opened up. I can now take a much lighter perspective on things.’ (P1)

‘I make plans, positive plans (…) [Before the DBS] I would not have dreamed about setting up an online store, or maybe I would have dreamed about it, but then like ‘that will never work out anyway’. But now I think: ‘oh, I can give it try.’ (P3)

‘I found myself picking up the phone, and even saying yes to an invitation. Just like that. After I hung up, I thought: ‘what have I done?’ (…) But then I thought: ‘so what?’ (P4)

‘I found myself doing things without thinking. That is pretty scary.’ (P10)

‘I know I actually have a spontaneous nature. (…) And I think I am getting closer now to who I really am.’ (P5)

‘I can enjoy the things that I do more. Before it was only obligations, now it is sometimes even fun, you know.’ (P3)

‘I have changed, yes. I have come more out of my shell, so to speak. I can enjoy life more than before.’ (P6)

‘I am more laconic.’ (P1)

‘My world has become so much larger. Before, I just lived in a cocoon of compulsions.’ (P14)

‘And than I feel that, it is incredible, I feel that my fear is decreasing, so then I am not having a cup of coffee with a psychiatrist, but with another human being!’ (P13)

As these quotations from the interviews illustrate, the effects of DBS on the experiences of OCD patients are global and thorough. Indeed, it seems warranted to say that the patients' way of being in the world has changed. So how can we understand these experiences? Can we discern a pattern among these experiences? Is there one central effect that in turn results in the other effects? For example, is changed *mood* the pivotal factor? The first effects patients generally report are effects on their mood: being less anxious and more open. Are these first effects also the most fundamental? Or is rather the changed *sense of agency* the hinge on which all other effects turn? Patients report more spontaneous actions, less excessive thinking, and a tendency to make plans, all effects that are related to a normalized sense of agency. They seem to rely more on their own actions and perceptions. Or should we even regard a changed *sense of self* as the key effect?

All of these elements seem to play a role. So how do they go together? In the next section we will propose an encompassing model in order to try to integrate and relate these various changes.

## An enactive affordance-based model

From a phenomenological perspective, psychiatric disorders such as OCD can be understood as a different way of being in the world. Similarly, we can understand the effects of DBS on the patients' experiences in terms of changes in their way of being in the world^6^. We propose to flesh out this, admittedly somewhat vague, notion of being in the world in four aspects: (1) person; (2) world; (3) characteristics of the person-world interactions; (4) existential stance toward these (1), (2), and (3).

Such an analysis is a tricky business, as the notion of a different way of being in the world is precisely meant to emphasize the *holistic* character of disorders. That is, disorders are not regarded as a coincidental heap of a number of independent symptoms: there is a *coherence* to these symptoms. Our analysis is not meant to deny or undermine this coherence: rather, it offers a means to develop a better understanding of what such a different way of being in the world amounts to. Spelling out various aspects helps to structure and relate the patients' diversity of experiences.

As the notion of being in the world already conveys, it is the interrelatedness of person and world that is at stake. Now, drawing on insights from enactivism and ecological psychology, we can develop a renewed perspective on what such dynamic person-world interactions amount to—one that fits with the (neuro)biological dimension of psychiatric disorders^7^. Enactivism (Varela et al., 1991; Thompson, 2007; Stewart et al., 2010) offers a dynamical systems perspective on the fundamental relatedness (or “coupling” in enactive terms) between person and world.

The focus on *dynamics* implies the relevance of taking into account the development and (mutually) reinforcing loops of coupled processes. And it is precisely such loops, gone awry, that we have seen to play an important role in the development of OCD, such as the hyper-reflexivity trap that we described. Regarding person and world as an integrated complex *system* implies a thoroughly relational perspective. This fits well with insights from ecological psychology, notably the concept of responsiveness to affordances (Gibson, 1979; Chemero, 2003; Rietveld, 2012a).

Taking the person-world interactions as central also opens the way to investigate the different ways in which this interaction takes place. That is, apart from investigating the world and the person pole of the interaction, respectively, we can categorize different *characteristics* of these dynamic interactions. Finally, we add the “existential stance” to the picture, that is, the acknowledgement of the fact that we can evaluatively relate to what we do and who we are—a crucial element in psychiatric disorders and their treatment. We will now discuss these four aspects of the person-world interaction in turn and show how they capture the various phenomenological changes that OCD patients experience through DBS treatment.

## World: a field of affordances

The experience of oneself and the experience of the world are two sides of one interaction, and in any situation, the focus can be on either. For instance, when I focus on the world, the tea beside me can attract me; it can invite me to take a sip; but when I focus on myself, I can feel I am thirsty. The self-description and the world-description point at different aspects of the same phenomenon, they just differ in focus. Another example: I can tell you that I feel hopeful, or I can confidently describe my plans for the future.

As the phenomenological psychiatrist Van den Berg writes: “Our world is not primarily a conglomeration of objects that can be described scientifically. Our world is our home, a realization of subjectivity.” (p. 39–40). Consequently, “when the psychiatric patient tells what his world looks like, he states, without detours and without mistakes, what he is like” (p. 46). Our experience of the world reflects our needs, wishes, insecurities, in sum, all that we care about—plus our own history of previous interactions. Thus, how I experience the world does not only depend on what is in fact out there, but it also depends on what I notice: what is *salient* to me. And this will in turn depend on two things: on my concerns (my current needs and interests and what I generally care about), and on my abilities (e.g., whether I perceive the steep face of the rock as accessible or not will depend on my climbing abilities). In short: how we perceive the world depends on what we can do and on what we care about doing.

This idea of the fundamental intertwinement of world and individual was first proposed by Von Uexküll (Von Uexküll, 1920) in his notion of the “*Gestaltkreis*.” Gibson (1979) later introduced the notion of “affordances.” Affordances refer to the possibilities for action provided to us by the environment (Gibson, 1979; Reed, 1996; Chemero, 2003). It is a truly relational notion: in the case of human beings, affordances are relations between aspects of the environment and available abilities of the individuals within a form of life, (cf. Chemero, 2003; Kiverstein and Rietveld, 2012). Which of the many available affordances a particular individual is responsive to in the particular situation, i.e., what invites, repels, or attracts the individual, depends both upon the individual's abilities and concerns (i.e., needs, interests, preferences), and on what the environment offers (Rietveld, 2008). We are surrounded by many affordances: a cup affords drinking from (and throwing at somebody, if need be), a phone affords calling someone, books afford reading, people afford talking to, and so on. We are immersed in and responsive to a whole *field* of affordances.

We distinguish between the *landscape* of affordances and a *field* of affordances. The *landscape* of affordances refers to all the possibilities for action that are open to a specific *form of life* and depend on the abilities available to this form of life. In our human case this notably includes socio-cultural practices. The landscape of affordances thus describes the so-called “ecological niche” of a form of life. A particular aspect of the environment, say a tree, can play a role in the landscape of affordances of multiple forms of life. Von Uexküll (Von Uexküll, 1920) gives the famous example of an oak tree: for a rabbit it affords digging a hole between its roots, to a woodworm it provides food, for a person it could afford shelter from sun or rain, or cutting. The *field* of affordances refers to the relevant possibilities for action that a particular *individual* is responsive to in a concrete situation, depending on the individual's abilities and concerns. The field of affordances is thus a situation-specific, individual “excerpt” of the general landscape of affordances.

We propose that the changed world that our OCD patients describe can be fleshed out in terms of changes in their field of relevant affordances. We can distinguish three dimensions to this field: the “width” refers to the broadness of the scope of affordances that one perceives. This dimension relates to having a choice or action options. The “depth” of the field refers to the temporal aspect: one not only perceives the affordances that are immediately present here and now, but one is also pre-reflectively aware of future plans and possibilities for action: the affordances on the horizon that one is responsive to, so to speak. This temporal horizon reflects our *anticipatory* affordance-responsiveness. Lastly, the “height” of each of the affordances refers to the relevance or importance of the affordances that one is responsive to, i.e., to the experienced solicitation or affective allure. This dimension of relevance and salience relates to motivation^8^.

**Figure 1:**
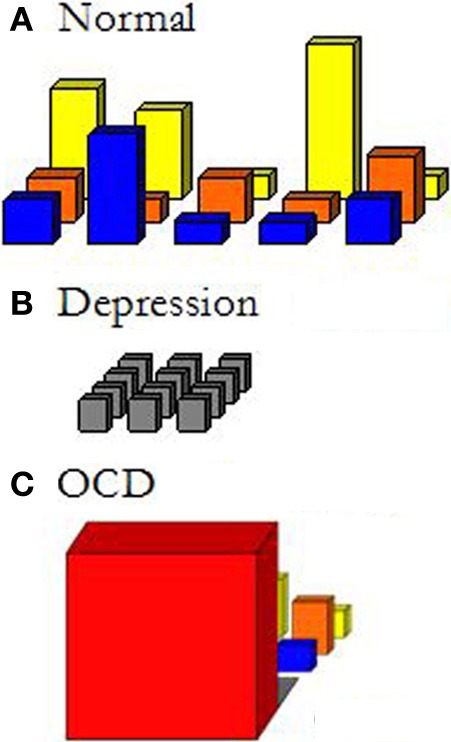
**Sketch of different fields of relevant affordances**.

Figure 1 gives a schematic depiction of different fields of relevant affordances.

To the extent that either our concerns or the environment change, the field of relevant affordances changes too. It is thus a *dynamic* field in all three dimensions: the scope of available affordances at a particular place that are taken into account by the person changes; the time span of the horizon of affordances changes; and there will be shifts in our concerns and, related to that, in the relative salience or relevance of the available affordances.

This notion of the field of relevant affordances allows us to make the phenomenological characterization of psychiatric disorders as changed ways of being in the world more concrete. For example, Figure 1B depicts the field of relevant affordances of a depressed patient. In contrast to the normally extended and differentiated field in Figure 1A, nothing stands out anymore; it is all the same gray, unattractive world that one is surrounded by. And this will go on and on endlessly: nothing sunny can be expected from the future either. We can depict the field of patients suffering from OCD, on the other hand, as extremely narrowed down to just the immediate affordance of what HAS to be done NOW (Figure 1C). Before any other relevant affordance may announce itself, completing the compulsion has first priority. First I need to thoroughly wash my hands, before I can even think of doing something else. The field of OCD is very much a field of fear or anxiety: fear narrows down the focus to just what is feared, and fear takes primacy over all other perceptions and actions. Psychiatric disorders can affect all three dimensions.

### Effects of DBS on the field of affordances

We can now take another look at some of the patients' experiences and see how we can understand them as changes in their respective fields of affordances.

‘I find that I can go for groceries much more easily, that I am not afraid to enter shops anymore.’ (P4)

‘The change in my mood, in my feeling of hope and courage, is very noticeable in my urge to make plans. (…) To think beyond where you are at the moment.’ (P1)

‘A large part of my daily life is now filled with social contact, talking to people, making plans, and just trying to get these off the ground.’ (P4)

The structure of the field of relevant affordances changes in all three respects as a result of DBS treatment: (a) the patients' interest in possibilities for action broadens, which is reflected in the increased width of the field of affordances that are taken into account by the patient; (b) the patients orient themselves on a larger time scale, which is reflected in increased depth; and (c) their detection of relevance becomes more adequate, that is, more in line with the totality of their concerns, which means that differentiation of affordances in terms of relevance increases. The first two aspects can be summarized as a growing ability to look beyond the here and now. Using the terminology of Heidegger (Heidegger, 1927/1978), we can say that they have recaptured the “*Entwurf*” side of their existence: the ability to relate not just to the *actual*, but also to the *possible*. Patients refer to having hope again, to making plans for the future, to being more broadly interested. When patients speak of feeling “open,” of “seeing possibilities” again, of realizing that they “can do” things, and of “seeing perspective,” this conveys the double movement that they open up to the world and the world opens up to them.

With regard to the third aspect, the relevance of the available affordances, patients report both more indifference and more involvement. It turns out that they can be more indifferent to objects and situations that used to draw all their attention (such as dirty dishes, potential sources of bacteria, etc.), and take an interest again in the things that they care about (e.g., in going out with friends and family). In short, we can say that their field of affordances now reflects their concerns beyond the mere urge to reduce the strong tension and anxiety. That is, their field of affordances now reflects what really matters to them once the distortion of anxiety is lifted. When we picture the red cubicle as a schematic structure of the field of affordances of OCD patients, we can picture how that red cubicle shrinks, and a more diverse and balanced field of affordances emerges from behind it, so to speak. Importantly, patients do not consider their interests to be new: they had always wanted to go horse-riding, or to study psychology, or to play in a band—it is just that before this was impossible due to their disorder and had slowly become unimaginable.

## Self-experience

This second aspect puts the focus on the person in the person-world interaction: it refers to the person's, typically pre-reflective, experience of herself in the interaction. To take our example of thirst: when focusing on the world-aspect, i.e., the field of affordances, the cup of tea shows up as highly salient and attractive to us. The self-experience side of this interaction is feeling thirsty. The field of affordances reflects this experience: it is the other side of the coin, so to speak. This aspect encompasses moods, feelings,^9^ and self-experience in general.

### Effects of DBS on the patients' self-experience

‘I dare to speak up for myself more. (…) That is a whole new dimension. People are surprised sometimes: “she has become a fierce lady”.’ (P2)

‘I am less anxious, and that also expresses itself bodily. (…) I don't get into a panic that often anymore; I am better able to find solutions’ (P5)

‘With the right settings, I am in a better mood. I can immediately notice that. It's… my breathing is calmer and more relaxed. (…) I feel less tense and so, well, I have less the need to do these compulsive actions’ (P3)

‘After the DBS I have become calmer, I don't panic so easily anymore’ (P5)

‘I dare to take risks now. I have become much more daring, more impulsive also.’ (P2)

‘I don't dwell upon things as long as I used to’ (P5)

One of the most common reports of our patients is that when DBS is effective, they experience a lessening of their feelings of tension and anxiety. Many patients also report an increase in self-esteem and feeling equal instead of inferior to other people. They speak out more, express themselves more, are more assertive. Many patients report changes in their social interactions: they feel more like interacting with others.

These experiences could well be related: the balance between anxiety and trust seems to shift toward the latter. Patients recount that they feel more confident, and they dare to do things again. This is reflected by the fact that they see more possibilities for action: the broadness of their field of affordances widens. Whereas being anxious entails withdrawing oneself, patients now speak of being more open to the world, including being open to other people.

## Characteristics of person-world interactions

So far, we have fleshed out the two sides of the person-world interactions. We introduced the notion of a field of affordances to depict the world as experienced by the person. The other side of this interaction is the person's experience of herself while responding to this field. Apart from these two sides to the interaction, we can distinguish a third element, namely the *way in which* persons relate to the world. This aspect refers to the characteristics or modes of relating, the “how” of the interaction.

This dimension of our model aims to capture the changes that patients experience with regard to the way in which they relate to the world. Their experiences point to changes in at least four different characteristics. First of all, one's intentionality can be primarily outward or inward directed. Is one's focus on what one is doing, or more inward directed, occupied by one's feelings and thoughts? Secondly, the interaction of the person with her world can be more or less flexible. This refers to the extent in which one is responsive to other possible actions. Can one easily switch between different affordances? A third characteristic already came to the fore in our discussion of the phenomenology of OCD, namely the amount of reflexive attention that one pays to one's action. Is the action performed unthinkingly, or rather with deliberate attention? Lastly, there is an affective dimension to the way in which one relates. For instance, are things done in a stressed or rather in a relaxed way?

These characteristics of the interaction are of course intimately connected with both self-experience and the field of affordances. In general, one's self-experience will likely (come to) reflect the way in which one interacts, and vice versa. For example, if one is much quicker to respond and has a less deliberating way of dealing with the world, one will probably experience oneself as more spontaneous. The same goes for the structure of the field of affordances: that will (in time) reflect one's way of relating as well. For instance, when one is rigidly focused on one task, one may lose sight of all the other tasks that one should also take care of. In this case the broadness and the horizon of the field of affordances will shrink.

The extent to which one pays deliberate attention to what one does may, in the long run, also have consequences for the field of affordances. We already introduced the hyper-reflexivity-trap to refer to the unsettling effect of paying too much conscious attention to what one does. In reaction to this unsettling effect, OCD patients may restrict their life world to just those actions that they can control. Inversely, a continual imbalance in the other direction, namely too little deliberation and reflexive awareness, will also affect the structure of the field of affordances. If one would never step back to reflect and only relate unthinkingly to the present situation, one would not be able to pursue more distal goals and direct one's efforts accordingly. The horizon of the field of affordances would then shorten to the immediate here and now.

### Effects of DBS on the characteristics of person-world interactions

‘I dare to take more risks’ (P5)

‘I dare to do things again’ (P2)

‘I am somewhat calmer now: I can keep my attention focused on my work instead of the compulsions’ (P3)

‘I used to have more control. Now my actions go faster than my thoughts. (…) My actions now just go automatically, instead of how I used to do it.’ (P10)

‘What I did not used to dare, the things that I did not even dare to begin with, I started doing them. I still found it scary, I was just as afraid of it, but I *did* it anyway.’ (P2)

‘I am better able to put things into perspective. Not so black and white anymore, but more looking at things from both sides, so more grey, you know. (…) I am not so quick to judge anymore (…) but I stay careful who I can trust and who not’ (P5)

‘The intensity, the anxiety, and the amount of compulsions have diminished. (…) as if it is crumbling away in all aspects’ (P4)

We can see that patients are (a) more outward directed, (b) more flexible, (c) acting more unreflectively, and (d) in a less stressed way. Thus, as a result of DBS-treatment, not only has their field of affordances changed, and their self-experience, but also their *manner of attaining grip* on this field.

With regard to the first characteristic, patients report a shift from more inward to more outward directedness, and related to that, an increased openness to available affordances. The world of obsessions and compulsions is unavoidably a very self-focused world. As long as the experienced tension is so strong, all other things have to wait. The less consumed the patients are by their obsessions and compulsions, the more space opens up for other interests and for attention to other people. Here too, there are looping effects operative: the increase in openness results in more interactions with the world, and this can in turn have a positive, “pulling” effect on the patients. Patients for instance engage in a sport club, and the expectance of the other members motivates them to show up even if things are going less well. Friends are more inclined to call, when they find that the person now indeed answers their calls.

The second characteristic is increased flexibility, which shows itself in various ways. Patients for instance report that they are more easily distracted from their obsessions and compulsions. But the flexibility also refers to “cognitive” flexibility, such as the flexibility to change one's opinion, or to be less inclined to think only in black and white (P5). The increase in creativity that some patients report, too, can be understood as a more flexible, associative way of relating to the world; an increased playfulness or openness to unconventional affordances.

With regard to the third characteristic, there is a shift from deliberate steering to more spontaneous, unreflective engagement. The balance between reflexive awareness, and deliberation on the one hand and unreflective immersion in action on the other, has normalized. As we noted earlier, this is an important characteristic of the phenomenology of OCD. The spontaneous actions also include the spontaneous diminishing of compulsive behaviors. That is, patients may simply forget to perform some of their rituals—and sometimes they do not even notice it until someone else points it out to them.

And lastly, patients experience less tension and stress: even when they still perform some compulsions the intensity of the tension has reduced.

## Existential stance: evaluation of self-world interactions

### Existential stance

So far, we have distinguished three aspects of the person-world interactions. First, we fleshed out the world-aspect in terms of a field of relevant affordances. Secondly, we looked at the person-aspect, e.g., the side of self-experience, including moods and feelings. Thirdly, we have distinguished various characteristics of the person-world interactions. Now, we want to introduce the fourth and last element: the existential stance. The existential stance refers to the person's evaluative relation to her world and to herself.

We do not just experience things, but we can also be *aware* of having these experiences, and we can *relate* to our experiences. We can take a stance on events in the world, and on other people. And we can take a stance on ourselves: on what we do and think and feel. We can be ashamed of present or past wrongs, we can be proud of ourselves, we can lie and feel guilty about lying, and we can dread things in the future or look forward to them. Just as the world does not appear neutral to us, but is full of things that attract or repel us, our interactions are not neutral to us either. That is, our stance-taking is an *evaluative* stance-taking.

Due to our capacity to reflect, we unavoidably relate to ourselves and the world around us. We can even relate to things that are not present: we relate to our past and future, and we can relate to imagined scenarios and events. The German philosopher Plessner (Plessner, 1981) calls this our ex-centric position: we do not coincide with our present situation but we can take a stance on it and thus obtain some degree of freedom and possibilities for change.

From a structural perspective, the existential stance can be seen as an extra “loop” in the system: not only does a person interact with her (social) environment, she also relates to these interactions and to the part she plays in them. The existential stance refers to this *second-order relation*. Thus, whereas the field of affordances refers to the first-order perception or experience of the world and self-experience refers to the first-order experience of oneself, the existential stance refers to the person's second-order relation to these primary aspects of the person-world interaction^10^.

Even though this second-order relation presupposes the ability to reflect, to detach oneself from the here and now, this does not imply that such a stance can only have the form of an explicit judgment or deliberate reflection. In fact, our stance on things is usually rather *implicit* in the way we behave. For example, the fact that I value friendship is manifest in my deeds. When I call a friend that I have not spoken to in a while, I am simply complying with my interest in contact with my friends. I am not explicitly putting my values into practice: it is rather that my field of affordances reflects what I care about. It may even be the case that I am not aware of how important my friends actually are to me, until someone comes along and points out that I spend a considerable amount of my time talking to friends, and thinking about them, and so on.

An important part of this existential relation is the stance we take toward ourselves. This includes implicit and explicit evaluations of who we are and who we want to be, what we consider to be our virtues and our vices. For people suffering from psychiatric disorders, this relatedness to oneself also includes a stance on their illness. Is the disorder considered an alien element that has just struck one out of the blue? Or is it on the contrary difficult to draw a line between what is part of who one is (e.g., perfectionist, or boisterous) and what might be part of a disorder (e.g., OCD, or ADHD)?

The existential dimension is highly relevant in psychiatry, both with respect to the etiology as well as the treatment of psychiatric disorders. The way in which patients relate to themselves and their situation may be an inherent part of the disorder itself. For instance, in the case of anxiety disorders, patients may suffer most from the fear of the possibility to get another panic attack. To make things even more complicated, psychiatric disorders can also affect patients' stance-taking. For example, part of being depressed consists of having no hope for future change and of a distorted perception of the past. Second order feelings of both shame and guilt often also constitute negative spirals. They stand in the way of social contact, while isolation generally worsens the situation. Both shame and guilt considerably contribute to the patients' suffering and feelings of loneliness as well. Moreover, feelings of shame may prevent patients from seeking help, whilst their condition worsens.

With regard to the other aspects we have distinguished, it is worth noting that the existential stance can bear upon any of these. With regard to the third aspect, one can take a stance on the way in which one interacts with the world, for instance one can enjoy and endorse one's more spontaneous way of responding to the world. And where the second aspect refers to the first-order self-experience, the existential stance refers to the evaluation of that self-experience: one can for example blame oneself for being down again. The first aspect, the field of affordances, reflects the world as experienced. The existential stance refers to the fact that one can also think about and assess one's situation from a more observer's perspective. In OCD this difference between the first-order experience of the world and the second-order reflective stance on the world is striking. For, as we already noted, OCD patients may experience all kinds of events as threatening even though they are well aware of the irrational nature of their fears and compulsive actions. From such an observer's perspective patients may *know* that there are other possible actions available to them (they are aware of the richer *landscape* of affordances), but their experience of tension narrows down their field of relevant affordances to just the compulsion that needs to be done first.

### Effects of DBS on the existential stance

‘I did not quite notice the changes at first, other people pointed me to what was happening. And only then I started to think and realized what had changed’ (P5)

‘I am much closer now to who I really am.’ (P5)

‘I found myself doing things without thinking. That is pretty scary.’ (P10)

‘I lashed out to the children. I reacted a bit too severe. That does not fit me.’ (P10)

‘My wife pointed out to me that the casing that I built around the pond looked a bit sloppy. I had a look, and she was right! I would never ever deliver sloppy work. It always had to be perfect. And now, I saw it was sloppy, and you know what? I did not even care!’ (P14)

‘I am more laconic.’ (P1)

‘The increase in libido was a side-effect. That is really not who I am.’ (P1)

‘Now I think: things will work out somehow. But before, nothing would ‘just work out’.’ (P4)

‘I can have much more normal contact with others now. My daughter or son-in-law call and invite me to a birthday party, or ask if they can come by at my place. Yes, and then without thinking about it, I just say yes. Because: I want to come, but then I realize: what am I saying? I really have to get used to myself, you know. Those excuses always, never being able to make any appointments… and then I just say ‘yes, sounds good’. And I think: Is that me? It is a bit strange, I have to get used to myself too.’ (P4)

The existential stance refers to the patients' evaluative relation to their changed way of being in the world. In the case of our patients, one of the central issues concerns their evaluation whether or not a certain change suits them. Changes may be endorsed, i.e., considered as contributing to becoming more oneself, or they may rather be regarded as mere side-effects. For example, the increase in libido is a common change, and some patients welcome this as a restoring of their “natural” libido that got lost as a result of the disease, whereas other patients clearly distance themselves from it and regard it as a side-effect. Similarly, for some patients, the experience of doing things unthinkingly is a bit scary, others, however, are happy about their spontaneous reactions and feel they have become more themselves.

Assessment of what does and does not suit one's “identity” is of course complicated. As several patients remarked: who knows who you would have been if you had not developed OCD in your youth or childhood? One patient (P14) said that he now finds himself doing things in a similar way as his father used to do them. That strengthens his intuition that these changes indeed allow him to be more himself.

Not only the patients themselves wrestle with these questions: so do their loved ones, and their therapists. Partners may have a different assessment of the changes than the patients themselves have. For instance, a patient may feel much better and more himself, whereas his partner considers him to be have become overly assertive and ruthless. The difficulty is that partners often only know their partner with OCD. Besides, within the relationship partners have somehow found a way to deal with the disorder, and have established certain shared patterns of interaction. The fast and profound changes the patient goes through can be unsettling for the partner as well: their world changes too. And when patients and their partners differ in their assessment of the changes, this poses a difficulty for the therapist, as she needs to decide to what extent the patient and the partner are able to give an accurate evaluation.

Another issue concerns the patients' stance on having an implanted device. Here too, patients differ in their assessment. Most patients have no problem with it, or even regard it as a part of their body, “in the same way that a new hip becomes part of one's body as well,” as one patient formulated it. Others feel uncomfortable about it, either because they are concerned about their dependency on the continuing care of the hospital or because they feel somewhat ashamed. A patient recounted that she is very careful about informing people about the DBS after she found out that some of her neighbors refer to her as “the cyborg”^11^.

The existential stance plays a double role in the effects of DBS treatment on the phenomenology of OCD patients. On the one hand, patients evaluate the changes they experience due to DBS treatment, with all the ensuing influences. But, interestingly, the patients' evaluative stance may itself change through DBS. Patients especially take a more laid back stance on themselves and their world. They report that they have become less perfectionist and that they actually endorse this change (like the patient who made a casing for the pond). Or patients not only care less about what others might think of them, but also embrace this more laconic attitude.

### Summary: four aspects, one way of being in the world

The four aspects presented in our model (the field of affordances, self-experience, the characteristics of the person-world interactions, and the existential stance) increase our insight in the phenomenon of a changed way of being in the world. It provides a structure to analyze the complex, global changes that OCD patients experience during DBS treatment. As we have stressed, these aspects really go together in the global way of being in the world. Take for instance this quote:

‘You have lived your life aside of the world, all those years, decades. I have had days that I was so turned inwards, continuously doing my rituals. And then I was lying in bed and heard at the radio that it had been a rainy day. And I thought to myself, was it rainy today? I had no idea. And your gaze has passed the windows of your house a thousand times, but you don't notice it, there is no outside world anymore. (…) But then I caught myself really listening to the news and I realized this might be a sign of a small, beginning, change. You start to read the newspaper, and you start to talk to people, say what they think of the financial crisis or something. Yes, it becomes more and more your own world.’ (P4)

This quote shows how the aspects cohere, how it all fits within a unitary way of being in the world. The patient's way of being in the world has changed: the narrow field of affordances of a life filled with compulsions gives way to an opening up to the world (aspect 1). Instead of his inward directedness and constant deliberation on how to avoid getting “contaminated,” he now reacts spontaneously (aspect 3). We can say he regains his unreflective responsiveness to relevant possibilities for action, such as picking up the ringing phone, or listening to the news or to what other people say. These affordances regain their relevance and solicit action again (aspect 1). His noticing of the changes and interpreting them as positive signs is an instance of the evaluative or existential stance (aspect 4). P4 describes how he increasingly participates in our shared world, and how fulfilling this is for him in contrast to the loneliness of his small, solitary, compulsion driven world (aspect 2). Later on he says: “I still have problems, but these are *ordinary* problems, that everybody has, such as a lack of money. I am so happy to have ordinary problems now.”

## Brain and person

We saw that treatment with DBS can induce profound changes in the patients' way of being in the world. What does this imply with regard to the relation between brain and personhood? If electrical stimulation of a specific brain area can bring about such profound phenomenological changes, does that mean that we “are our brains” as some neuroscientists (Crick, 1995) like to state?

Let us start by noting that the treatment of our patients is not limited to manipulating the brain only. DBS treatment encompasses more than just turning on the device. It usually takes months to find the optimal settings of the electrodes, and during this time the patient comes to the department weekly or bi-weekly and is intensively monitored. Moreover, patients also take part in CBT. It appears to be the combination of DBS and CBT which makes the treatment all the more effective. DBS diminishes the patients' experience of anxiety and tension and many improvements follow naturally from that. Double-blind experiments in which DBS is turned either on or off indeed show that DBS is effective (Denys et al., 2010). But CBT (or other psychotherapies) often play a crucial role in recovery as well. For years, or even decades, the patients' lives have revolved around their compulsions. And even if the felt urge to perform compulsions diminishes through DBS, it still takes effort and practice to shape a new way of living. The therapists help them not only to reduce the time spend on their compulsions, but also to discover and develop alternative skills and activities that they now finally have time to engage in. Many patients report that the success of treatment consists in precisely the combination of DBS and CBT: the DBS provides the crucial thrust that enables to them to successfully engage in CBT. “It is hard work,” says a patient, referring to CBT, “but I am so glad that I am now finally able to do it.”

Still, we have shown that DBS can bring about profound immediate phenomenological changes. Does the effectiveness of DBS commit us to a neuro-reductionist account that goes against the idea behind neurophenomenology (Varela and Thompson, 2003; Thompson, 2007)? It does not. Our findings support an enactive, systemic perspective rather than a reductionist one.

The first thing we need to clarify is the relation between neural processes and experience. If one starts from the assumption of two ontologically different processes that subsequently need to be related, one is likely to end up with either a dualist or a reductionist view. From an enactive perspective, however, we rather start from the whole complex system of a person interacting with the landscape of affordances. The experiential dimension and the neural dimension are different *excerpts* from this complex system—at different levels of resolution (De Haan, Submitted manuscript). Experience depends on the whole system of a person interacting with her world in a concrete situation. The brain too functions only within this entire system. When we look at neural processes, we are in fact zooming in on what happens in the brain during these person-world interactions^12^.

A change in my experience implies or encompasses a change in my neural processes. Interfering in the brain IS interfering in the larger system. Because the brain is part of the person-world system, changing the brain implies changing the person-world system. Now, a complex system of a person interacting with her world offers many possible ways of interfering. For instance, DBS targets specific neural networks, CBT targets specific cognitive and behavioral habits, psycho-analysis targets specific insights at the existential dimension. Even though such interventions can never be completely isolated from the context (like for instance the treatment-relationship), all of these interventions do have a specific target, and can be recognized as a major causal force. But more often then not, there will be a whole mesh of direct and indirect influences. Also it will often take a while before changes at a local level build up to changes at a more global level of the system. Such threshold phenomena go both ways: it may also take time for global changes to get engrained at a local level. The complexity and reciprocity of these processes fit much better with an enactive, systemic approach than with a reductionist one.

From an enactive perspective of the complex person-world system the effectiveness of DBS is not surprising. Given that the brain is such an important part of the system, as a crucial connecting organ (Fuchs, 2011a), it makes perfect sense that interventions at the neural level have the potential to have far-reaching effects. These effects pertain to the system as a whole—and may include the person's experiences. The effects of DBS thus in no way warrant a reductionist equation of persons and their brains. Our research project rather fits in with a non-reductionist and non-dualist approach to neurophenomenology. The particular role of the brain is put in a broader perspective which includes not just the person, but also her interaction with the environment. The notion of responsiveness to a field of affordances can help to flesh out the crucial role of our interactions with the world for our phenomenology.

## Conclusion

We started our paper with the observation that the global and thorough changes that OCD patients experience during DBS treatment are insufficiently captured by the available psychiatric scales. These scales typically focus on symptoms only and do not capture any other changes, such as changes in self-experience, social interactions, existential stance, the feeling of openness toward the world, and the way in which patients react to what the world has on offer. Such changes are, however, fundamental to understand the impact of DBS, as well as to assess the patients' well-being—which in turn is an important factor in the determination of changes in DBS-parameter settings and treatment more generally. We noted that the patients' whole way of being in the world seems to have changed. Our aim was to capture the changes in the patients' phenomenology and make sense of the broad range of changes they report. For that we have introduced a model that fleshes out the dynamic interactions between person and world in four aspects. We proposed to specify the patients' world in terms of a field of affordances, with the three dimensions of broadness of scope (“width” of the field), temporal horizon (“depth”), and salience or relevance of the perceived affordances (“height”). The second aspect is the person-side of the interaction, that is, the patients' self-experience, notably their moods and feelings. Thirdly, we have pointed to the different characteristics of the way in which patients relate to the world. And lastly, the existential stance refers to the stance that patients take toward the changes they experience: the second-order evaluative relation to their interactions and themselves.

In this paper we have limited ourselves to exploring the phenomenological changes of a very particular group of patients. As we have mentioned before, an important characteristic of DBS treatment is that, compared to other forms of treatment, the effects of DBS can be very quick and direct. For the aim of developing our model this provides a unique opportunity, because it allows us to witness “*in vivo*” the rapid phenomenological changes from disorder to recovery. Given that phenomenological changes occur through other forms of treatment as well and with patients suffering from different psychiatric disorders, we hope that our model may turn out to be of broader use. Future qualitative and quantitative research will have to verify and possibly extend our way of specifying the notion of being in the world. Such an explicit phenomenological model may help to assign to the patients' experiences the fundamental role in psychiatric practice that they deserve.

### Conflict of interest statement

The DBS devices used in this study were provided by Medtronic Inc. Dr. Denys was supported by an unrestricted investigator-initiated research grant by Medtronic Inc.

## Footnotes

^1^Accounting for the importance of patients' experiences does not deny the importance of the biological and socio-cultural dimension of psychiatric disorders. We rather acknowledge the complexity of psychiatric disorders than try to reduce them to only one or two dimensions. Our model fits with a broader enactive framework for psychiatry, one that also encompasses the (neuro-)biological and socio-cultural dimensions of psychiatric disorders, see: (De Haan, Submitted manuscript).

^2^Affordances are the possibilities for action provided to us by the environment (Gibson, 1979). We will explain this notion and our use of it in An Enactive Affordance-Based Model of this article.

^3^Although this may be a common course of the illness, it does not capture all cases (Denys, 2011).

^4^OCD patients experience tension, they explicitly acknowledge that they want to have grip on things, and they strive for things to “feel right.” However, they apparently experience this tension much sooner (e.g., with regard to situations that to other people appear harmless) and also more intensely than others generally do. What could be the cause of this deviation? To date, there is no conclusive answer to the question where this tension comes from. And there need not be just one answer: there could be various causal trajectories at play in different patients [cf. (Borsboom et al., 2011)].

^5^Research on memory shows that patients with OCD do not have memory impairments *per se*, but rather *distrust* their memory: it is thus the *attitude towards* their memory that is affected. Interestingly, the same happens to normal controls who are instructed to repeatedly check their tasks. The checking thus augments the feeling of insecurity. See: (van den Hout and Kindt, 2003).

^6^Some of these changes will be inherent to the process of recovery *per se*, such as the shift from inward to outward directedness that we will describe later on. Our aim is to shed light upon what happens as a result of DBS treatment, but this does not imply that all these changes are unique for this specific form of treatment. Like we mentioned before, DBS does provide a unique opportunity to witness in vivo processes that are otherwise likely to take much more time.

^7^We will not pursue this issue here. For more on how an enactive approach can relate the various dimensions of psychiatric disorders see: (De Haan, Submitted manuscript).

^8^This dimension of relevance or salience needs further unpacking. As one of our reviewers remarked, there are many different ways in which the perceived environment can solicit activity. Something can be important because it is dangerous and needs to be avoided, or because it is highly attractive and pulling us in. This means we need to further explore in future work the relation between affective salience of the affordance, and whether something presents itself as an attraction or rather as an “avoidance.” In our schematic depiction we have used colours to add some differentiation to the dimension of experienced relevance. The height refers to the *intensity* of the relevance, whereas the colours are meant to reveal something about the *affective* salience of the affordance. An affordance can be highly relevant because it seems life threatening (say, red), or because it is related to the person we are in love with (say, pink). We thank the reviewer for highlighting the need for further research on this topic.

^9^Several psychologists have argued for distinguishing a world- and a self-aspect to emotions (Lambie and Marcel, 2002; Frijda, 2007): there is a way in which the *world appears* when you are in a specific emotional state and there is a way in which *you feel* when you are in that state. In our terminology, one can focus on the world-aspect of an emotion by looking at the changes it brings about in the field of affordances (e.g. what action possibilities show up, what we regard as salient, etc.). Or one can focus on the person-aspect, that is, the person's self-experience.

^10^It is important to note that this existential relation does not come over and above our ‘basic’ interactions with the world: it rather changes the whole person-world system—our way of being in the world. For instance, even our most basic biological acts such as eating, drinking, defecating, sleeping, and having sex are thoroughly shaped by our specific socio-cultural practices and mean much more to us than only the satisfaction of a biological urge (De Haan, Submitted manuscript).

^11^For more on the stance people may take on “artificial” elements in their bodies, see: (Slatman, forthcoming).

^12^Such an enactive, systemic perspective has important implications for how we conceive of the causality between neural and experiential processes, see: (De Haan, Submitted manuscript).
